# Readers and their roles: Evidence from readers of contemporary fiction in the Netherlands

**DOI:** 10.1371/journal.pone.0201157

**Published:** 2018-07-26

**Authors:** Allen Riddell, Karina van Dalen-Oskam

**Affiliations:** 1 School of Informatics, Computing, and Engineering, Indiana University-Bloomington, Bloomington, Indiana, United States of America; 2 Department of Literary Studies, Huygens Institute for the History of the Netherlands, Amsterdam, Netherlands; 3 Department of Modern Dutch Literature, University of Amsterdam, Amsterdam, Netherlands; Aarhus Universitet, DENMARK

## Abstract

Reading serves many ends. Some readers report that works of fiction provide an imaginative escape from the rigors of life, others report reading in order to be intellectually challenged. While various characterizations of readers’ engagement with prose fiction have been proposed, few have been checked using representative samples of readers. Our research reports on reader self-descriptions observed in a representative sample of 501 adults in the Netherlands. Reader self-descriptions exhibit regularities, with certain self-descriptions predicting others. Contrary to existing theories which posit two types of readers characterized by non-overlapping concerns (identifying readers and distanced readers), we find that while some readers attend to plot structure or read in order to be intellectually challenged, reader self-descriptions overlap more than received theories predict. We hypothesize that some readers have cultivated more reading techniques than others, with educated or experienced readers tending to report deriving additional experiences from reading.

## Introduction

In her study of romance novel readers in the United States, Radway [1] documents a surprising variety of reasons offered for reading. Some readers reported that reading provided a pleasurable imaginative escape. Others, however, described the act of reading, with minimal reference to the “content” of literary works, primarily as an activity which licenses a temporary physical withdraw from work and family obligations [1]. A general typology of how readers approach literary works is offered by von Heydebrand and Winko [2]. They identify two “reader roles” (*Leserrollen*), distinguishing between “identifying reading” and “distanced reading” (*das identifikatorische und das distanzierte Lesen*) [2]. Readers using identifying reading (“identifying readers”) seek to empathize with or otherwise recognize the main character and the problems the character encounters [3]. By contrast, readers using distanced reading (“distanced readers”) are characterized by their attention to the “formal characteristics of texts” (*formale Eigenschaften der Texte*). Distanced readers also disapprove of making direct associations between the fictional text and personal experiences.

The purpose of the present study is to test this characterization of how readers tend to encounter literary works. To accomplish this we draw on a representative sample of 501 reader from the Dutch National Reader Survey. We ask if the respondents in the Survey describe themselves as readers in a manner consistent with the two reader roles identified by von Heydebrand and Winko. We find that the readers’ responses are incompatible with the account offered by von Heydebrand and Winko. Respondents who describe their reading in terms consistent with distanced reading are more likely to also describe themselves in terms consistent with identifying reading.

## Previous research on reader roles

Much has been written about readers and reading, also of fiction reading specifically. In this section we survey work relevant to the theory of reader roles we seek to test.

Before proceeding, we note a distinction which is observed by some but not all of previously published research. A distinction should be made between *which* works of fiction readers choose to read and *how* they approach reading. While this is frequently a distinction without a difference—the works a reader selects may be predicted with high probability from a self-description of how they read—the distinction is essential. Two readers, one who describes themself as enjoying paying attention to formal elements of the text and another who refuses such a description, may read the same book, likely attending to different parts or different aspects of the literary text as they read. A useful analogy, taken from Hennion [4], is the situation where two people, one a wine connoisseur and the other a non-connoisseur, drink from the same bottle of wine. In such a setting, we are not surprised if the two report experiencing different things. The theory of reader roles which we aim to test, as the name “reader role” suggests, is more concerned with how readers think about themselves as readers than with the particular kinds of works they tend to read.

### Theories of reading

The characterization of readers by von Heydebrand and Winko, has already been briefly introduced. Their identification of two distinct, non-overlapping reader roles is part of a larger project aimed at modeling the process of literary evaluation [3]. They partition readers into two types of readers, the identifying and the distanced reader. As identifying readers seek a personal relationship with the text, their reception of the literary work varies with the reader: “[Identifying readers] relate primarily to the [work’s] contents and associate what they read directly with their own experiences and problems” (*Sie beziehen sich in erster Linie auf deren Inhalt und beziehen das Gelesene direkt auf eigene Erfahrungen und Probleme*). Whereas identifying readers are characterized by their identification with the main character(s) in the text, distanced readers are characterized by (1) their focus on aesthetic conventions and formal features of the text and (2) by the tendency to deprecate the association between the fictional text and personal experience characteristic of identifying reading [2]. From the perspective of the distanced reader role, identifying reading is “negatively sanctioned” (*negativ sanktioniert*) and judged to be “inadequate” (*defizitär*) [2].

While von Heydebrand and Winko imply that their characterizations of reader roles is intended to support the empirical study of literary evaluations, they have not carried out empirical studies themselves.

Felski [5] offers a broader perspective on reading in the widely-read and comprehensive *Uses of Literature*. Felski distinguishes among four “modes of textual engagement”: reading involving “a logic of recognition”, in which the work of art expands or extends a reader’s self-understanding; reading dwelling on the enchantment of intense aesthetic experience; reading furnishing the reader with maps for understanding social reality; and reading engaging with texts with the aim of being shocked and having commonsense assumptions challenged. Felski states that these four modes of textual engagement are not tied to intrinsic literary properties or to independent psychological states, but “denote multi-leveled interactions between texts and readers that are irreducible to their separate parts” [5]. According to Felski, literature is likely valued for different, even incommensurable, reasons [5].

The four modes as described by Felski (2008) are enlightening. They merit further investigation and formal modeling. In practice, we found formulating a strategy to check Felski’s descriptive hypotheses challenging.

### 0.1 Reading and social differentiation

Studies of how and what people read have frequently appeared in the context of research on social differentiation. Kraaykamp and Dijkstra [6] report on two experiments dealing with social differentiation in book reading preferences in the Netherlands. Their aim was to explain why readers from backgrounds associated with higher social status are more likely to display a preference for more complex and prestigious books. They performed an experiment in which they asked readers to rank a list of books and genres according to their complexity and literary prestige. The results of this ranking were used as a measurement of the reading preferences of the participants. Kraaykamp and Dijkstra go on to investigate to what extent the background of the readers predicted differences in these preferences. For this, they made use of additional information about the respondents, such as reading habits in their home environment, education, current social situation, and reading habits of friends. They found that education is useful in predicting which books readers read, as is the current social status of the respondent. Readers from households with more leisure time and higher income tended to read more complex books. Furthermore, readers tended to favor complex genres more when they indicated they had a highly educated best friend. Kraaykamp and Dijkstra judged these results a confirmation of the idea that a preference for complex works of fiction may be seen as linked to group-specific tastes.

The ranking experiment used by Kraaykamp and Dijkstra was inspired by Nell [7]. Nell reports on his research on individuals he labels ‘ludic readers’, people who read at least one book a week. His respondents are mostly from South Africa. His research combines different experiments and methods, such as the ranking experiment, interviews, lab sessions measuring reading time, respiration rate, and cardiac activity of respondents. Nell pays special attention to the selection of reading material, wishing to find out how ludic readers select titles that they expect will give them pleasure. The ranking experiment shows that readers have different opinions about books they read for pleasure and books known for literary prestige. A difficult book generally is seen as probably having literary value, whereas an enjoyable book is felt to be low quality. While Nell acknowledges that literary merit and difficulty should be viewed as independent characteristics of a work, in practice he reports that they are intertwined and that readers sometimes mistake one for the other. Nell looks for an explanation to the Protestant Ethic and to an association between difficulty and virtue.

Nell never discusses reader roles in his own research. He resists the idea that readers are either highbrow or lowbrow, labeling such a dichotomy an “elitist fallacy”. Nell is convinced that sophisticated readers have learned how to enjoy high-brow literature, but also that these readers continue, ‘on occasion and if their consciences allow them’, to find pleasure in reading narratives that are much more stereotyped, about such ‘low-brow’ things as quests of heroes and heroines and so forth.

## Methods

### Dutch National Reader Survey

The data which we analyze comes from a panel survey of 501 individuals conducted between August 20, 2013 and August 26, 2013 (hereafter “panel survey”). The panel survey is a component of the larger Dutch National Reader Survey. The Dutch National Reader Survey is, in turn, part of The Riddle of Literary Quality project which is funded by the Royal Netherlands Academy of Arts and Sciences (KNAW). Respondents were drawn from the ca. 50,000 members of the inVotes panel maintained by the market research company No Ties BV.

A version of the survey was developed and tested in December 2012. The results from the pilot version informed the design of a large-scale online survey which was featured in major newspapers and available to the general public between March 3, 2013 until September 27, 2013. The version of the survey used in the online survey was then used with the inVotes panel. The data analyzed here are from 501 individuals in the panel who responded to this survey. The survey was developed with the assistance of Erica Nagelhout of Nagelhout.MRS and carried out by No Ties BV, a Dutch market research company. Both Nagelhout.MRS and No Ties BV are members of the Dutch industry organization for market research, MOA: Expertise Center for Marketing-Insights, Onderzoek & Analytics (MOA). As such, they abide by the MOA’s Gedragscode voor onderzoek en statistiek (Code of Conduct for Research and Statistics). The code specifies procedures for the protection and use of personal data (https://www.moaweb.nl/codes-standards/richtlijnen/). For the panel survey we use, respondents were drawn from the ca. 50,000 members of the inVotes panel gathered and maintained by No Ties BV. The panel members are 18 years and older and have consented to be part of the panel; they are recruited from various websites and receive a small reimbursement for their work. No Ties BV maintains panel members’ privacy. They ensure that other parties do not have access to panel members’ data and that these data are never provided to other parties (https://home.noties.nl/home/invotes/). Panel members invited to take part in The Riddle of Literary Quality survey were free to quit at any moment they wished. For these reasons, no approval of an institutional review board was sought when the survey was conducted. In February 2018, we submitted the study of which the panel survey is part, The Riddle of Literary Quality, to the Ethics Committee of the Faculty of Humanities of the University of Amsterdam (http://aihr.uva.nl/about-aihr/ethics-committee/ethics-committee.html) for an evaluation of the survey after the event. The Committee has evaluated the submission for the study The Riddle of Literary Quality (Dossier 2018-10). The Procedure for Ethical Evaluation of the Faculty of Humanities of the University of Amsterdam states that research has to be submitted before the research is carried out. Therefore it cannot be approved afterwards. The Committee states, however, that had the research been submitted before it was carried out, it would have been approved by the Committee.

The composition of the population of respondents is calibrated according age, sex, social class, and the five Nielsen-regions in the Netherlands: (1) Amsterdam, Rotterdam, and The Hague, (2) Noord-Holland, Zuid-Holland and Utrecht, (3) Groningen, Friesland and Drenthe, (4) Overijssel, Gelderland and Flevoland, and (5) Zeeland, Noord-Brabant and Limburg. The calibration follows guidelines established by the Markt Onderzoek Associatie (MOA) for all research agencies and research purposes, in cooperation with Centraal Bureau voor de Statistiek (CBS), a Dutch government institution.

### Reader role questions

Questions inspired by von Heydebrand and Winko’s account of reader roles (“reader role questions”) were incorporated into the panel survey. These questions allowed respondents to characterize their reading in terms taken from von Heydebrand and Winko’s typology of reader roles. Each question offers respondents a claim with which they are asked to agree or disagree. The six questions are evenly divided between those proposing a self-description compatible with the identifying reader role and those proposing a self-description compatible with the distanced reader role.

Questions were developed to test the reader roles theory without requiring that respondents be familiar with the precise formulation of the typology. For example, one of the statements with which identifying readers were expected to endorse concerns whether or not the story is a “true story.” The reasoning here is that stories billing themselves as based on facts reliably aim for realism. Realism is important for any reader seeking to associate events in a narrative with experiences in their own life. Distanced readers, by contrast, would be indifferent as to whether events were tied to real events.

#### Identifying reader role questions

The following three questions were designed to allow readers who see themselves as adopting an identifying relationship with works of fiction to characterize their reading:

- I read novels to discover new worlds and unknown ages. (*Ik lees romans om nieuwe werelden en onbekende tijdperken te leren kennen*).
- I like novels which are based on true stories. (*Ik hou van waargebeurde romans*).
- I want to be swept away by a novel. (*Ik wil graag meegesleept worden door een roman*).

#### Distanced reader role questions

The following three questions were designed to allow readers who see themselves as adopting a distanced reader role to characterize their reading:

- I like to explore the deeper layers of a novel. (*Ik ga graag op zoek naar de diepere lagen van een roman*).
- I like to think about the plot structure of a novel. (*Ik denk graag na over de opbouw van een roman*).
- I read novels in order to be challenged intellectually. (*Ik lees romans om verstandelijk uitgedaagd te worden*).

Respondents were asked if they “agreed or disagreed with the description” (*Kunt u steeds aangeven in hoeverre u het eens of oneens bent met deze stellingen?*). Respondents answered using a five-point scale ranging from “totally disagree” (*helemaal mee oneens*) to “totally agree” (*helemaal mee eens*). In our analysis, responses were transformed into a binary scale by coding the two responses indicating agreement (“totally agree” and “agree”) as “agree” and coding other responses as “not agree”. A sixth option of “Don’t know” (*weet niet*) was also available to respondents. Responses of “Don’t know” were rare (1.5% of the 2,202 responses). In models these responses are handled by treating the particular question as not having been answered by the respondent. Elsewhere (e.g., in tables) we treat these responses as “not agree”.

Of the 501 respondents who participated in the panel survey, 367 (73%) said that they read prose fiction (Table 1). This percentage aligns with comparable figures from the United States. In 2002 roughly 72 in 100 adults in the United States report having read a novel, short story, poem, or play (other than those required by work or school) in the last year. In the United States women are also more likely to report reading fiction than men [8].

**Table 1:** Demographic characteristics of respondents in the survey. Percentages are percentages within rows.

		Does not read fiction	Reads fiction	N
Man	< Univ. degree	49%	51%	148
	≥ Univ. degree	26%	74%	98
Woman	< Univ. degree	22%	78%	144
	≥ Univ. degree	5%	95%	111
All		27%	73%	501

As the reader role questions elicit self-descriptions of readers of prose fiction, only the 367 respondents who reported reading fiction were asked to respond to the six reader role questions.

## Ideal point model

We use an ideal point model to analyze responses to the reader role questions. The ideal point model is frequently used in political science to study legislators’ and judges’ votes. An individual’s “ideal point” is a coordinate in some space and is typically interpreted as capturing their (latent) preferences. In the case of models of political and judicial behavior the latent point typically inhabits a one dimensional latent space and is interpreted as associated with the ideological disposition of the voter [9–11]. The adequacy of the ideal point model is evaluated in terms of its ability to predict observed votes. In the present context, readers’ ideal points will be interpreted as latent dispositions which predict their answers to the reader role questions. The ideal point model is used to test two things. First, the model is used to assess whether or not there is any pattern in individuals’ responses to the reader role questions. Second, assuming there are reliable patterns, the model is used to check if the observed patterns of responses are consistent with the identifying and distanced reader typology offered by von Heydebrand and Winko.

The ideal point model is derived from the two-parameter logistic item-response model, also called a Rasch model [12]. (The two-parameter logistic (2PL) item-response model is widely used in educational testing). The model assumes that each respondent and each question occupy a point in a one dimensional latent space and that the relative position of a respondent’s point and a question’s point predict how a respondent will answer a question. By using a shared space for questions and respondents, the model is able to capture patterns in responses. Respondents associated with similar positions will tend to answer questions the same way. The models considered here are standard models and their formal presentation is available in greater detail elsewhere [11].

To justify the use of the ideal point model we also consider a simple, baseline model (“Model 1”) which models an individual’s responses to the reader role questions as independent of each other. Model 1 has one parameter for each question and these parameters express the probability with which each question is likely to elicit agreement. The ideal point model, described in detail below, will be labeled “Model 2”. We also consider an extension to the ideal point model. This third model, “Model 3”, adds two parameters to the ideal point model to allow for occasional haphazard responses by participants. (Such unpredictable responses are known as “guessing” in the context of related models used in educational testing).

**Fig 1:**
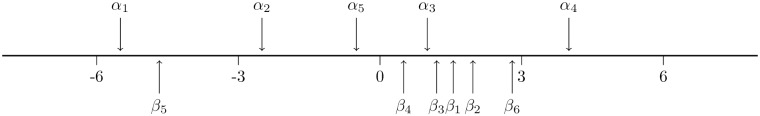
Illustration of ideal point model. Illustration of ideal point model with five respondents and six questions. If a respondent’s latent position *α*_*j*_ is greater than the position of the question *β*_*k*_ then the probability of an “agree” response (*y*_*jk*_ = 1) is greater than 0.5. (This assumes the question’s discrimination parameter *γ*_*k*_ is positive).

Model 2, the standard ideal point model, models responses using question-specific parameters and respondent-specific parameters. Responses are represented in the following way. The *K* = 6 questions are answered by *J* = 367 individuals. If person *j* agrees with the statement proposed by question *k*, we record this as *y*_*jk*_ = 1. If they do not agree, *y*_*jk*_ = 0. The probability that person *j* agrees with question *k* is given by

$$\text{Pr}\left(y_{jk}=1\right)={\text{logit}}^{-1}\left(\gamma_{k}\left(\alpha_{j}-\beta_{k}\right)\right)$$

where logit^−1^ is the inverse logit function, *α*_*j*_ is the latent position of respondent *j*, *β*_*k*_ is the position of the question *k*, and *γ*_*k*_ is the “discrimination” of question *k*. If *γ*_*k*_ = 0, the question does not “discriminate” in any way, as Pr(*y*_*jk*_ = 1) = 0.5. (In the educational or psychological testing setting, *α*_*j*_ is often called the “ability” of the respondent and *β*_*k*_ the “difficulty” of the question). If the magnitude *γ*_*k*_ is high, the probability increases (or decreases) relatively rapidly as the latent position of the respondent and question diverge. In ideal point research the sign of *γ*_*k*_ indicates the specific response (“agree” or not) given the latent position of the respondent and question. For example, if *α*_*j*_ = 4, *β*_*k*_ = 1, and *γ*_*k*_ = 1 then the probability of person *j* agreeing with the statement posed by question *k* correctly is logit^−1^(1 × (4 − 1)) = 0.95. Fig 1 illustrates how the *α* and *β* values might be distributed on a line.

Model 3 expands the ideal point model to allow for accidental responses. We consider this expanded model out of concern that logistic models such as the one considered here are not robust to the presence of extreme values [13]. An extreme value in this setting would be, for example, a case where *y*_*jk*_ = 0 despite a high predicted probability of an “agree” response (i.e., logit^−1^(*γ*_*k*_(*α*_*j*_ − *β*_*k*_)) is near one). Model 3 adds two parameters which model the probability of an accidental response.

Estimating parameters for Model 2 and Model 3 is complicated by the problem of non-identifiability. One can, for example, multiply all parameter estimates by -1 and arrive at the same probability for an observed “agree” response. Following Bafumi [11], we resolve this problem by modeling the prior distributions for the *α* parameters using side information. The prior distribution for the *α*_*j*_ parameter associated with each respondent varies according to whether or not the respondent reports reading only fiction (i.e., they do not read non-fiction works). Apart from identifying the probability distribution, this prior distribution does not influence parameter estimates.

We estimate parameters for all three models using Stan, software for Bayesian analysis [14]. Source code and data used in the analysis are available in S1 File. Expressing the ideal point model in the domain-specific language used by Stan was made considerably easier thanks to documentation accompanying [15].

## Results

### Summary measures of responses to reader role questions

The reader role questions were presented to respondents in the form of statements describing them as readers, statements with which respondents were asked to agree or disagree. Some of the statements rarely elicited agreement. Others were endorsed frequently. Table 2 shows the aggregate frequency with which respondents agree with the reader role questions. The statement with which respondents most frequently agreed is “I want to be swept away by a novel” (84% agree). The statement which elicited the least aggregate agreement is “I read novels in order to be challenged intellectually” (30% agree).

**Table 2:** Aggregate agreement with reader role questions. Percentages are percentages within rows. The full text of the statements appears in Section Reader role questions.

		new worlds	true stories	being swept away	deeper levels	plot structure	intellectual challenge	N
Man	< Univ. degree	37%	39%	75%	25%	24%	28%	75
	≥ Univ. degree	40%	36%	82%	29%	38%	42%	73
Woman	< Univ. degree	34%	47%	86%	34%	28%	25%	113
	≥ Univ. degree	39%	42%	92%	40%	42%	28%	106
All		37%	42%	84%	33%	33%	30%	367

**Fig 2:**
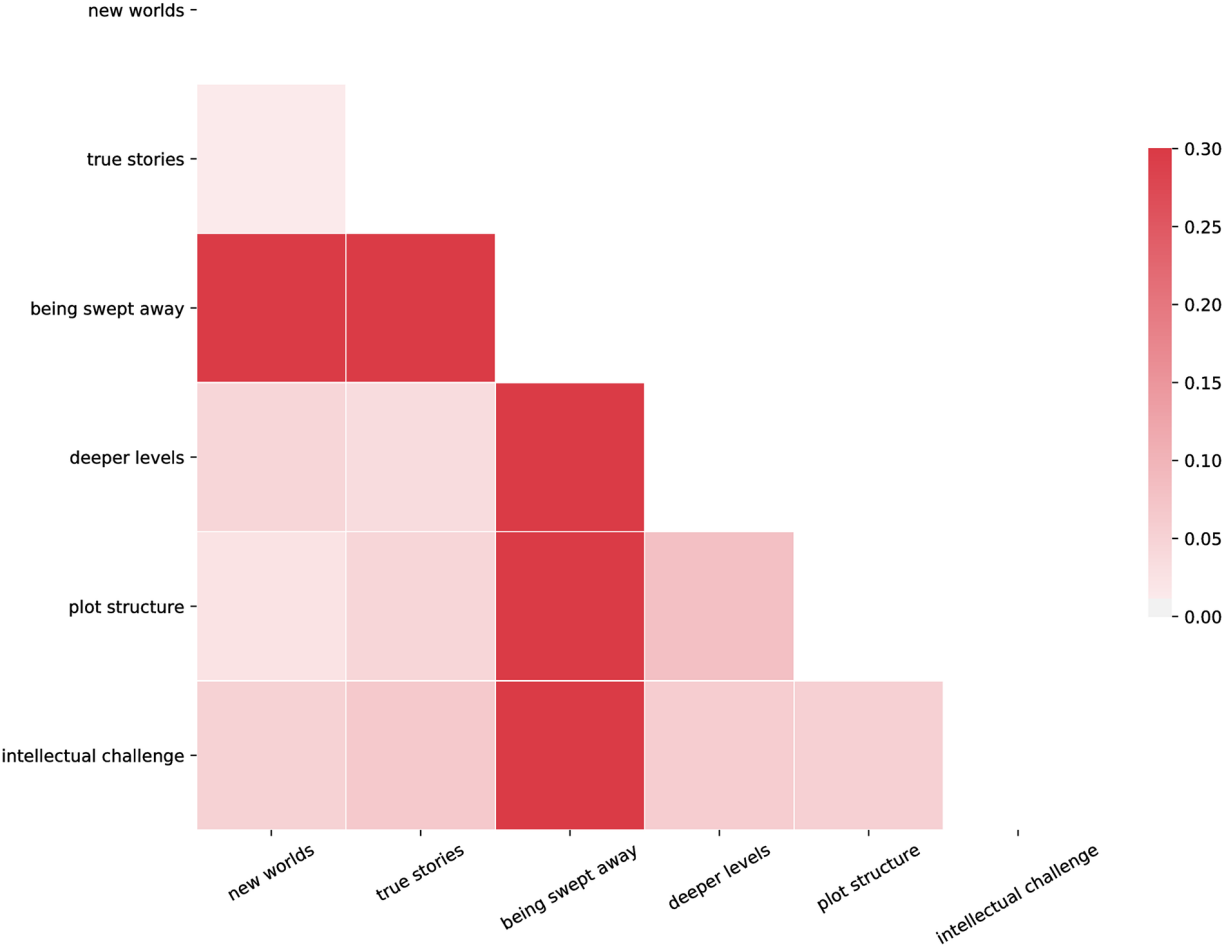
Empirical mutual information among responses to reader role questions. Mutual information among responses to reader role questions (*N* = 367). Mutual information is calculated using the empirical distribution of responses.

A preliminary appreciation of regularities in individual responses can be gained by considering the empirical mutual information among the responses (Fig 2). A mutual information of zero implies that two outcomes are independent. Comparing the mutual information between responses to the reader role questions provides a sense of how learning the response to one statement facilitates prediction of responses to other statements. For example, learning whether or not a respondent agreed with the “I read novels in order to be challenged intellectually” question tells us, on average, more about whether or not they agreed with the statement “I want to be swept away by a novel” than about their response to the statement “I like to think about the plot structure of a novel”.

## Responses to reader role questions

The ideal point model describes the observed data better than the model which assumes a reader’s responses are independent. An individual’s response to a reader role question tends to predict how they will respond to other questions. We find that if a reader agrees with any one of the distanced reader questions they tend to also agree with all the identifying reader questions. For example, readers who agree with the statement “I like to think about the plot structure of a novel” will tend to also agree with the statement “I want to be swept away by a novel.” This result appears to contradict the prediction we associate with von Heydebrand and Winko.

Table 3 summarizes our main result. To compare models, we use the widely applicable information criterion (WAIC) to estimate the leave-one-out predictive fit of models [16, 17]. WAIC takes into consideration the effective number of parameters used by a model as models with a greater number of parameters can be expected to fit the data better. Higher WAIC scores indicate a better fit with the observed data.

**Table 3:** Model comparison. Analysis of 2,170 individual responses to the reader role questions. $\hat{\text{lpd}}$ is the computed log pointwise predictive density, roughly analogous to the log-likelihood. ${\hat{p}}_{waic}$ is $\sum_{i=1}^{n}\;{\text{Var}}_{s=1}^{S}\text{log}p\left(y_{i}|\theta^{s}\right)$, the simulation-estimated effective number of parameters, analogous to *p*_*D*_ in the deviance information criterion (DIC). WAIC is $\hat{\text{lpd}}\;-\;{\hat{p}}_{waic}$. Calculations are based on 10,000 draws (40,000 draws, saving every 4th) after 40,000 iterations of warmup.

	Model 1	Model 2	Model 3
Description	Fixed probability for each question	Ideal point model	Ideal point model with guessing
$\hat{\text{lpd}}$	-1319	-946	-913
${\hat{p}}_{waic}$	6	214	254
WAIC	-1325	-1160	-1167

Our analysis shows that the ideal point model is more consistent with observed responses than the simpler model which assumes independent responses. As higher WAIC scores indicate, both Model 2 and Model 3 fit the observed data better than Model 1. Model 3, which includes parameters modeling guessing, is not associated with a higher WAIC score, suggesting that haphazard responses are not a significant concern. We use the results of Model 2 in the following discussion.

**Fig 3:**
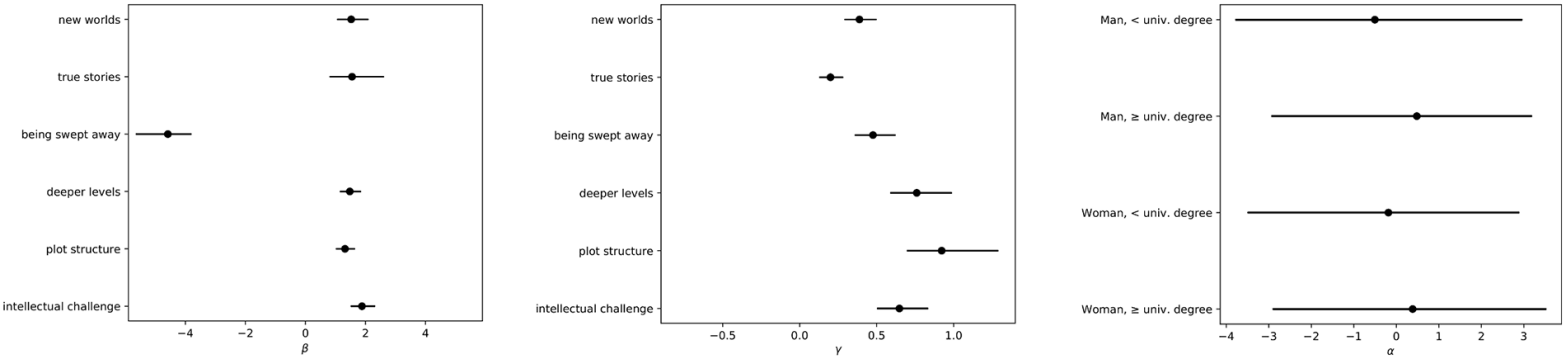
Parameter estimates for ideal point model of responses to reader role questions. The figure shows parameter estimates for ideal point model of responses to reader role questions (Model 2). The left-hand side figure shows the posterior distribution for the latent position, *β*_*k*_ for each reader role question. The center figure shows the posterior distribution for the discrimination, *γ*_*k*_, for each reader role question. The right-hand figure shows the posterior distribution of the latent positions, *α*, for subgroups of respondents. All intervals shown are 80% credible intervals.

The parameter estimates from Model 2 offer a narrative of the pattern of responses to the reader role questions. Fig 3 shows the inferred positions of the questions, the discrimination of each question, and the latent positions of the respondents by gender and education. Respondents who tend to agree with a reader role question with a value of *β*′ will tend to agree with all reader role questions associated with a value of *β* less than *β*′. (The discrimination parameters *γ* are uniformly positive.) For example, respondents predicted to agree with “I read novels in order to be challenged intellectually” (*β*_6_) will tend to, with probability greater than 0.5, agree with all the other statements. By contrast, knowing only that someone agrees with the statement “I want to be swept away by a novel”, one does not learn whether they agreed with the other statements. This is consistent with what we would expect having looked at Table 2: most people agree with this statement.

The question with the highest discrimination parameter is associated with the statement “I like to think about the plot structure of a novel.” Higher (positive) discrimination indicates that an “agree” answer to the question more reliably indicates that a respondent’s latent position is to the right (higher) than the question.

The patterns visible in the ideal point model fit are not the patterns that the theory of von Heydebrand and Winko would lead us to predict. Indeed we see the exact opposite. Those who answer in a way that suggests they are distanced readers tend to be more likely to also answer in ways that suggest they are identifying readers.

We do not claim to have identified a pattern which will hold for all contemporary readers of fiction. It may be that readers in the Netherlands are atypical in any number of ways. Readers of prose fiction in Germany or in other national situations may well conform to the typology advanced by von Heydebrand and Winko. An additional possibility we cannot discount—due to the size of our sample—is that certain, relatively small subpopulations of readers (e.g., professional literary critics, holders of degrees in literary studies) conform to the reader role typology.

## Discussion

We report on patterns in reader self-descriptions recorded in a representative sample of 501 readers of fiction in the Netherlands. Our main result is that reader self-descriptions exhibit regularities and these regularities appear to contradict at least one existing theory of how readers engage with fiction. Readers are not easy to partition into two non-overlapping classes: identifying readers who seek fiction which allows them to empathize or otherwise identify with the main character and distanced readers who are characterized by their tendency to attend to aesthetic conventions and formal characteristics of texts. Rather, we find that some readers describe themselves in terms of the identifying reader alone and some readers describe themselves in terms of the identifying reader and, moreover, report attending to formal characteristics of fictional works (e.g., plot structure).

In broad strokes, the responses to the reader role questions suggest that readers distinguish themselves not by expecting different things from the reading experience but in terms of how many different experiences they expect. Whereas some readers hope to be swept away by the narrative, others anticipate more, such as an interesting plot structure or intellectual challenges.

We speculate that a distinguishing characteristic of the readers who anticipate additional experiences is that they have cultivated more *reading techniques*. Readers deploying these techniques have different experiences when they read prose fiction. This conjecture is inspired by Schwarz [18], who offers a sociology of taste focused on different tasting techniques. These techniques tend to be costly to acquire and can, when they are used, influence the experience of the aesthetic object. For example, Schwarz discusses this techniques-centered sociology of taste in the context of art viewing and wine tasting, discussing how the deployment of techniques can both affect the experience of the art object and be, in its use, visible to others. In the case of wine tasting, Schwarz draws on Hennion [4] for a description of the range of “corporeal techniques” used by the experienced wine taster, including smelling the wine and, during the initial encounter with the wine, sipping a small amount of wine and attending carefully to its characteristic “feel” in the mouth. The use of these “micro-level tasting act[s]” mean that “people from different backgrounds often taste objects differently” [18].

Differential access to reading techniques, we propose, yields a situation consistent with the responses we observe in the panel survey, where people report anticipating different experiences from the encounter with prose fiction. We lack evidence of comparable, granular “reading techniques” being acquired or taught, but we suspect that many do exist and are frequently taught—often under the general heading of “close reading.” Substantiating this hypothesis will, however, require further study of reading in the wild.

## Conclusion

In this contribution we reported on reader self-descriptions recorded in 2013 from a representative sample of 501 residents of the Netherlands. We analyzed the respondents’ agreement with six statements, three of which aligned with an identifying reader role which could be described as reading for pleasure, escape, and identification, and three of which aligned with a distanced reader, who could be described as a “literary” reader, more likely to be in search of an aesthetic experience. We find that reader self-descriptions exhibit regularities, with certain self-descriptions predicting others. Contrary to the theory of two reader roles from which we departed (from von Heydebrand and Winko), we found that readers seem to distinguish themselves by how much they expect from their reading experience. Agreement with statements seen as describing the distanced reader (e.g., seeking intellectual challenge) predict a higher probability of agreement with statements seen as describing the identifying reader, in particular, wanting to be “swept away by a novel”.

Based on these results, we hypothesize that the differences between groups of readers may be associated with differences in the number or type of acquired reading techniques. This would fit with the observation that more educated readers derive more distinct experiences from reading and would therefore come closer to received expectations that formal training in reading literature plays an important role in establishing different kinds of readers.

## Supporting information

S1 File Source code and data. (ZIP)
